# Understanding Interactions Between Life Satisfaction and Genetic Predisposition on Risk of Alzheimer's Disease up to 14 Years Later: Findings From the UK Biobank

**DOI:** 10.1002/gps.70120

**Published:** 2025-06-29

**Authors:** Amber John, Roopal Desai, David Bartres‐Faz, Dorina Cadar, Darya Gaysina, Aida Suarez Gonzalez, Natalie L. Marchant, Emily Willroth, Marcus Richards, Rob Saunders, Joshua Stott

**Affiliations:** ^1^ Psychology Department University of Liverpool Liverpool UK; ^2^ ADAPT Lab Research Department of Clinical Educational and Health Psychology UCL London UK; ^3^ Faculty of Medicine and Health Sciences Institute of Neurosciences University of Barcelona and Institut d’Investigacions Biomèdiques August Pi i Sunyer (IDIBAPS) Barcelona Spain; ^4^ CEDAR Lab Department of Clinical Neuroscience Brighton and Sussex Medical School (BSMS) University of Sussex Brighton UK; ^5^ EDGE Lab School of Psychology University of Sussex Brighton UK; ^6^ Dementia Research Centre UCL London UK; ^7^ Division of Psychiatry UCL London UK; ^8^ Psychological & Brain Sciences Washington University in St. Louis St. Louis Washington USA; ^9^ MRC Unit for Lifelong Health and Ageing at UCL University College London London UK; ^10^ CORE Data Lab Research Department of Clinical Centre for Outcomes and Research Effectiveness Educational and Health Psychology UCL London UK

**Keywords:** Alzheimer's disease, life satisfaction, longitudinal, UK biobank

## Abstract

**Objectives:**

Previous research investigating associations between life satisfaction and risk of Alzheimer's disease (AD) has been mixed. This association may differ depending on genetic risk for AD. The aim of this study was to test interactions between life satisfaction and genetic predisposition on the future incidence of AD diagnosis.

**Methods:**

Data were used from 66,668 participants aged 60+ from the UK Biobank. Participants attended an assessment centre at baseline, and data were linked to hospital admissions data and death records up to 14 years later. Cox proportional hazards models were used to test interactions between life satisfaction and a polygenic risk score (PRS) for AD on incident AD diagnosis. Models were also run stratified by genetic risk for AD.

**Results:**

Models adjusted for age, sex, ethnicity, deprivation, education, and depression showed main effects of both life satisfaction (OR = 0.78, 95% CI = 0.68–0.90, *p* = 0.001) and the AD PRS (OR = 2.26, 95% CI = 2.12–2.40, *p* < 0.001) on incident AD. There was a significant interaction between the two (OR = 1.21, 95% CI = 1.09–1.35, *p* < 0.001). Stratified models showed that life satisfaction was associated with lower incident AD in the low, but not in the high genetic risk group (low: OR = 0.56, 95% CI = 0.42–0.75, *p* < 0.001; high: OR = 0.88, 95% CI = 0.75–1.04, *p* = 0.13).

**Conclusions:**

Results show that genetic risk for AD modified the relationship between life satisfaction and the risk of AD. This suggests that genetic risk may weaken associations between life satisfaction and AD risk. The findings clarify the mixed results of previous research on this topic and may contribute to more tailored approaches to the prevention of AD in the future.

## Summary

- Higher life satisfaction was linked to a lower risk of Alzheimer's disease over 14 years, even after adjusting for other factors.
- A polygenic risk score (PRS) for Alzheimer's was associated with an increased risk of developing the condition.
- There was a gene‐environment interaction, with stronger associations between life satisfaction and AD risk in those with low genetic risk.
- Life satisfaction may be associated with reduced AD risk, but this relationship is weaker in those with high genetic risk, suggesting a need for personalised prevention strategies.

## 1 Introduction

Alzheimer's disease (AD), the most common form of dementia, is a progressive neurological condition, which is characterised by cognitive decline and impaired daily functioning [1]. This condition affects approximately 55 million people across the world [2] and is associated with a range of personal, societal, and economic impacts [3]. In the absence of widely available treatments that can modify disease progression, understanding key risk and protective factors for AD is critical to contribute to risk reduction and prevention efforts.

Previous research has identified a range of potentially modifiable risk factors for AD, including health conditions (e.g., diabetes, hypertension, and traumatic brain injury), lifestyle and social factors (e.g., smoking, excessive alcohol, physical inactivity, and social isolation), socioeconomic factors (e.g., educational attainment) and environmental exposures (e.g., air pollution) [4]. Many of these risk factors have been tested across a range of different populations and datasets, including large‐scale studies such as the UK Biobank.

One potentially modifiable protective factor is life satisfaction, which can be defined as an individual's evaluation of their own life circumstances and quality [5]. However, relatively little research has focused on this construct in relation to AD. Previous research investigating associations between life satisfaction and risk of AD (and dementia more generally) has been mixed [6, 7], with some studies showing inverse associations [8, 9, 10], and others reporting no such association [11]. As such, associations between life satisfaction and AD risk remain underexplored, particularly in large population datasets such as the UK Biobank.

One explanation for mixed findings is that associations between life satisfaction and AD may differ depending on other key factors. For example, it is possible that life satisfaction may interact with genetic factors in the development of dementia. Gene‐environment interaction refers to the interplay between environmental factors and genetic variation, and the impact of this on human traits and health outcomes [12]. This is particularly relevant for complex conditions, such as dementia, which are likely linked with multiple interacting genetic, lifestyle, and environmental factors [13].

It is possible that the association between life satisfaction and risk of AD may differ depending on genetic risk for AD, but the current evidence for other modifiable risk factors is mixed. Several studies have reported that APOE genotype significantly modifies the association between smoking status and risk of dementia, whereby current smoking was associated with a higher risk of developing dementia amongst people who were not APOE‐ε4 allele carriers, but not in carriers [14, 15, 16], including in Alzheimer's disease specifically [14, 16]. By contrast, other studies have shown that modifiable risk factors (e.g., hypertension and overall healthy lifestyle) were associated with dementia, regardless of genetic risk [17, 18]. Finally, a further study showed an interaction between depression and APOE genotype on risk of Alzheimer's disease, in which the strongest associations were observed in participants with the APOE‐ε4 allele [19].

However, it is not yet known whether there is an interaction between life satisfaction and genetic predisposition on the risk of developing AD. This is important because it may help us to understand whether interventions to improve life satisfaction can contribute to AD risk reduction efforts, and who may benefit most from these interventions. The aim of this study was first to test associations between life satisfaction and AD diagnosis, and second to test whether this association differs depending on genetic predisposition.

## 2 Methods

### 2.1 Participants

Data were used from UK Biobank, a large prospective study of 503,316 people aged 40–69 at baseline. During 2006–2010, participants attended a baseline visit to assessment centres across the UK. Over the course of the study, participants provided a range of lifestyle, physical, health, and genetic data. In addition, data from the UK Biobank have been linked to healthcare records, including hospital admissions data (up to 2022) and death records (up to 2023). Participants under the age of 60 were excluded from the current analyses. This was because the incidence of AD increases substantially with age and is relatively uncommon before the age of 60. Participants who had a record of dementia other than AD over the follow‐up period (up to 14 years) were also excluded. AD is used as the outcome for this study, rather than all‐cause dementia, because the PRS is for AD.

### 2.2 Measures

#### 2.2.1 Diagnosis of Alzheimer's Disease (AD)

Diagnosis of AD was based on the UK Biobank algorithmically defined outcomes [20]. These were based on clinical codes to identify the earliest record of a range of health conditions and were developed and validated by the UK Biobank Outcome Adjudication Group, together with clinical experts. This algorithm uses data sources from UK Biobank baseline assessment data (i.e., self‐report), linked hospital admission data, and death register data. Self‐report data were not used for the AD diagnosis, because they applied to all‐cause dementia and cognitive impairment, not AD specifically. ICD‐9 and ICD‐10 codes used to identify AD across these sources have been published elsewhere [20]. One validation study reported an overall positive predictive value of 71.4% for the identification of participants with AD [21].

#### 2.2.2 Polygenic Risk Score (PRS) for Alzheimer's Disease

The AD PRS was available as part of the UK Biobank's broader release of polygenic scores for 53 different diseases and quantitative traits. A Bayesian approach was used to derive PRS values based on meta‐analysed summary statistics from external genome‐wide association studies (GWAS). The APOE region was excluded from the PRS in order to isolate polygenic contributions beyond this major risk locus. The construction of the PRS followed a consistent pipeline across all traits, including quality control, SNP selection, and weighting procedures. Detailed methodologies for how the PRS was derived are available online [22, 23]. Limitations of the PRS, such as ancestry‐specific performance and lack of rare variant inclusion, are discussed in the original publications. For this study, the pre‐calculated derived AD PRS provided by the UK Biobank were used.

#### 2.2.3 Life Satisfaction

Participants were asked to rate their level of satisfaction within different domains of their lives, including work, health, family, friendships, and finances. Response options ranged from 0 (extremely dissatisfied) to 5 (extremely satisfied). A mean score of the items was derived to create a global measure of life satisfaction. As participants in this sample were aged 60+, many had not completed the work question, likely due to retirement. For this reason, the work item was excluded from the global life satisfaction measure. The life satisfaction questions were introduced partway into fieldwork during April 2009, meaning that data were available for a smaller sample than the original baseline cohort. Although the measure of life satisfaction used is not a validated multi‐item scale, single‐item life satisfaction measures have demonstrated good validity in large‐scale epidemiological data [24].

#### 2.2.4 Covariates

Covariates were age, sex, ethnicity, deprivation, education, and depressive symptoms (a related but distinct construct to life satisfaction). All covariates were selected from the baseline assessment. Age was a continuous measure of chronological age at the time of baseline. Sex was a binary measure coded as ‘Male’ and ‘Female’. Ethnicity was a categorical variable coded as ‘White’, ‘Mixed’, ‘Asian or Asian British’, ‘Black or Black British’, ‘Chinese’, and ‘Other ethnic group’. Deprivation was assessed using the Townsend index based on the national census output areas corresponding to participants' postcodes. Education was measured based on the age at which the participant completed full‐time education. Participants were also asked about the frequency of depressed mood within the last 2 weeks, ranging from 0 (not at all) to 3 (nearly every day). Correlations between life satisfaction and depressive symptoms were checked to ensure inclusion of this covariate did not introduce issues of multicollinearity. There was a moderate correlation between the two (*r* = −0.34, *p* < 0.001), indicating multicollinearity was unlikely to cause problems in the statistical models.

### 2.3 Statistical Analyses

#### 2.3.1 Main Analyses

Cox proportional hazards models were used to test associations between life satisfaction and AD PRS, and diagnosis of AD. Time to event was measured in years from the date of attendance at the baseline assessment centre (number of days divided by 365). The analysis follow‐up period started from 1 year after the assessment centre date, to minimise the potential influence of undiagnosed AD at baseline. Participants were censored at the date of death or at the end date for which hospital inpatient data were available (England: 31st Oct 2022; Scotland: 31st Aug 2022; Wales: 31st May 2022). The proportional hazards assumption was checked using Schoenfeld residuals. Models were first run including life satisfaction and AD PRS as key predictors. Next, models were re‐run including the interactions for life satisfaction with AD PRS to test whether associations between this component of wellbeing and diagnosis of AD differ depending on genetic predisposition. For this, PRS scores were categorised into a binary variable based on the median (high vs. low). Where statistically significant interaction terms were observed, models were stratified by the binary PRS score to test whether there were significant associations between life satisfaction and risk of AD diagnosis within high and low PRS groups. For each set of analyses, two models were run: Model 1—Unadjusted; Model 2—Adjusted for all covariates (described in detail above).

#### 2.3.2 Supplementary Analyses

Supplementary analyses were run in which the follow‐up period started from 5 years after the date of attendance at the assessment centre. This was to further minimise any potential impact of undiagnosed or prodromal AD present at the time of the baseline on findings.

## 3 Results

### 3.1 Sample Descriptives and Missing Data

As the life satisfaction questions were only available from April 2009, this analysis was restricted to people who participated from that time (*N* = 94,283). Of this sample, 71,717 people had data available for life satisfaction at baseline and AD PRS. Of these, a further 4863 (6.78%) participants were excluded due to missing data on inpatient source for deriving data on censoring date and 186 (0.26%) participants were excluded due to an event or censor date within 1 year of the assessment centre attendance. This resulted in a sample size of 66,668 for unadjusted models. Of this sample, 21,560 (32.34%) had missing data on at least one covariate, resulting in a sample size of 45,108 in fully adjusted models (Supporting Information S1: Figure 1).

#### 3.1.1 Missing Data

The sample with complete data on all key variables and covariates (*N* = 45,108) was compared to the sample with missing data (*N* = 49,175). The sample with complete data was more likely to be female, older, from white ethnic backgrounds, and to have had more frequent depressive symptoms. It should be noted that despite statistically significant differences between the groups, effect sizes were very small (Cohen's *d* < 0.2 for all comparisons) (Supporting Information S1: Table 1).

#### 3.1.2 Sample Description

The overall sample descriptives are presented in Table 1. In summary, the average age of the sample at analytic baseline was 64.31 years (SD = 2.79), with a larger ratio of women compared to men (women: *N* = 24,218, 53.69%) and of people from white ethnic backgrounds compared to minority ethnic backgrounds (white ethnic backgrounds: *N* = 43,491, 96.42%).

**TABLE 1:** Sample descriptives.

Characteristic	Mean (SD) or *N* (%)
Age, mean (SD)	64.31 (2.79)
Sex, *N* (%)	
Female	24,218 (53.69)
Male	20,890 (46.31)
Ethnicity, *N* (%)	
White	43,491 (96.42)
Mixed	150 (0.33)
Asian/asian british	672 (1.49)
Black/black british	486 (1.08)
Chinese	77 (0.17)
Other	232 (0.51)
Deprivation, mean (SD)	−1.42 (2.82)
Age left education, mean (SD)	16.50 (2.32)
Depression, *N* (%)	
Not at all	36,765 (81.50)
Several days	6679 (14.81)
More than half the days	1095 (2.43)
Nearly every day	569 (1.26)
AD diagnosis, *N* (%)	
No	44,373 (98.37)
Yes	735 (1.63)
Years to AD diagnosis, mean (SD)	9.34 (2.75)
Life satisfaction, mean (SD)	3.59 (0.56)
AD PRS, mean (SD)	0.04 (0.99)

In total, 735 (1.63%) participants had a recorded diagnosis of AD over the follow‐up period. On average, AD was diagnosed 9.34 (SD = 2.75) years after the baseline (range = 1.04–13.32 years). Compared to the participants who did not develop AD over the follow‐up period, those who did had lower mean scores of life satisfaction (*t* = 3.26 df = 45,106, *p* = 0.001), and higher scores on AD PRS (*t* = 26.73, df = 45,106, *p* < 0.001) (Supporting Information S1: Table 2).

### 3.2 Associations Between Life Satisfaction and AD PRS With the Incidence of AD Diagnosis

The proportional hazards assumption was not violated (*χ*2 = 12.13, df = 14, *p* = 0.60). Results from Cox proportional hazards models showed that higher level of life satisfaction were significantly associated with lower incidence of AD diagnosis over the follow up period in both unadjusted (OR = 0.77, 95% CI = 0.69–0.86, *p* < 0.001) and fully adjusted (OR = 0.78, 95% CI = 0.68–0.90, *p* = 0.001) models. AD PRS was significantly positively associated with diagnosis of AD in unadjusted (OR = 2.21, 95% CI = 2.10–2.33, *p* < 0.001) and adjusted (OR = 2.26, 95% CI = 2.12–2.40, *p* < 0.001) models (Table 2).

**TABLE 2:** Associations between life satisfaction and AD PRS with incidence of AD diagnosis.

	Model 1: Unadjusted	Model 2: Fully adjusted^a^
	*N* = 66,668	*N* = 45,108
Life satisfaction	0.77 (0.69–0.86), < 0.001	0.78 (0.68–0.90), 0.001
AD PRS	2.21 (2.10–2.33), < 0.001	2.26 (2.12–2.40), < 0.001
Age	—	1.25 (1.22–1.28), < 0.001
Sex		
Female	—	*REF*
Male	—	1.10 (0.95–1.27), 0.22
Ethnicity		
White	—	*REF*
Mixed	—	1.38 (0.51–3.69), 0.52
Asian/asian british	—	0.92 (0.50–1.69), 0.79
Black/black british	—	1.32 (0.70–2.50), 0.40
Chinese	—	0.74 (0.10–5.25), 0.76
Other	—	0.66 (0.21–2.06), 0.48
Deprivation	—	1.04 (1.01–1.07), 0.003
Age left education	—	0.99 (0.95–1.02), 0.40
Depression		
Not at all	—	*REF*
Several days	—	1.15 (0.94–1.41), 0.19
More than half the days	—	1.40 (0.92–2.13), 0.11
Nearly every day	—	1.16 (0.61–2.20), 0.65

^a^ Proportional hazards assumption not violated: *χ*2 = 12.13, df = 14, *p* = 0.60.

### 3.3 Interactions Between Life Satisfaction and AD PRS on the Incidence of AD Diagnosis

There was a significant interaction between life satisfaction and AD PRS on incidence of future AD diagnosis in unadjusted (OR = 1.13, 95% CI = 1.04–1.24, *p* = 0.005) and adjusted (OR = 1.21, 95% CI = 1.09–1.35, *p* < 0.001) models, indicating an attenuated association between life satisfaction and future AD in people with higher genetic risk of AD (Table 3). Models were then stratified by the binary measure of AD PRS (high vs. low, based on median scores). Analyses in the low PRS group showed significant associations between life satisfaction and future AD (unadjusted: OR = 0.59, 95% CI = 0.47–0.74, *p* < 0.001; adjusted: OR = 0.56, 95% CI = 0.42–0.75, *p* < 0.001) (Table 4). Though there was a significant association between life satisfaction and AD in the high PRS group in unadjusted models (OR = 0.85, 95% CI = 0.75–0.96, *p* = 0.008), this did not persist after adjustment for key covariates (OR = 0.88, 95% CI = 0.75–1.04, *p* = 0.13) (Table 4). This attenuation was driven by the inclusion of depressive symptoms as a covariate.

**TABLE 3:** Interactions between life satisfaction with AD PRS on incidence of AD diagnosis.

	Model 1: Unadjusted	Model 2: Fully adjusted
	*N* = 66,668	*N* = 45,108
Life satisfaction	0.68 (0.60–0.78), < 0.001	0.65 (0.55–0.77), < 0.001
AD PRS	1.42 (1.04–1.95), 0.03	1.14 (0.78–1.67), 0.50
Life satisfaction × AD PRS	1.13 (1.04–1.24), 0.005	1.21 (1.09–1.35), < 0.001
Age	—	1.25 (1.22–1.28), < 0.001
Sex		
Female	—	*REF*
Male	—	1.10 (0.95–1.27), 0.22
Ethnicity		
White	—	*REF*
Mixed	—	1.35 (0.50–3.61), 0.55
Asian/asian british	—	0.93 (0.51–1.71), 0.83
Black/black british	—	1.31 (0.69–2.48), 0.41
Chinese	—	0.70 (0.10–5.00), 0.72
Other	—	0.65 (0.21–2.04), 0.46
Deprivation	—	1.04 (1.01–1.07), 0.003
Age left education	—	0.99 (0.95–1.02), 0.40
Depression		
Not at all	—	*REF*
Several days	—	1.14 (0.93–1.39), 0.22
More than half the days	—	1.39 (0.91–2.11), 0.13
Nearly every day	—	1.05 (0.55–2.00), 0.88

**TABLE 4:** Associations between life satisfaction and incidence of AD diagnosis stratified by PRS score.

	Low PRS		High PRS	
	Model 1: Unadjusted	Model 2: Fully adjusted	Model 1: Unadjusted	Model 2: Fully adjusted
	*N* = 33,464	*N* = 22,605	*N* = 33,204	*N* = 22,503
Life satisfaction	0.59 (0.47–0.74), < 0.001	0.56 (0.42–0.75), < 0.001	0.85 (0.75–0.96), 0.008	0.88 (0.75–1.04), 0.13
Age	—	1.27 (1.20–1.35), < 0.001	—	1.23 (1.20–1.27), < 0.001
Sex				
Female	—	*REF*	—	*REF*
Male	—	1.18 (0.86–1.61), 0.31	—	1.09 (0.92–1.28), 0.32
Ethnicity				
White	—	*REF*	—	*REF*
Mixed	—	—	—	1.76 (0.66–4.71), 0.26
Asian/asian british	—	1.32 (0.48–3.66), 0.59	—	0.81 (0.38–1.73), 0.59
Black/black british	—	0.75 (0.18–3.10), 0.69	—	1.32 (0.64–2.69), 0.45
Chinese	—	—	—	1.46 (0.20–10.43), 0.71
Other	—	—	—	0.99 (0.32–3.09), 0.98
Deprivation	—	1.05 (0.99–1.11), 0.08	—	1.03 (1.00–1.06), 0.02
Age left education	—	0.96 (0.90–1.04), 0.34	—	0.99 (0.95–1.03), 0.61
Depression				
Not at all	—	*REF*	—	*REF*
Several days	—	1.01 (0.64–1.57), 0.98	—	1.20 (0.96–1.52), 0.11
More than half the days	—	1.08 (0.43–2.69), 0.87	—	1.46 (0.91–2.35), 0.11
Nearly every day	—	1.18 (0.41–3.36), 0.76	—	0.95 (0.42–2.16), 0.91

### 3.4 Supplementary Analyses

Analyses were re‐run using a follow‐up period that began 5 years after the date of assessment centre attendance, to further minimise the impact of undiagnosed AD present at baseline. All models were fully consistent with those presented in the main results section (Supporting Information S1: Tables 3 and 4).

## 4 Discussion

Our findings showed that life satisfaction and genetic predisposition for AD were each independently associated with future incidence of AD diagnosis up to 14 years later. Models including interaction terms and stratified models also revealed that genetic risk significantly modified the relationship between life satisfaction and future AD. Specifically, life satisfaction was associated with lower future incidence of AD in the low genetic risk group, but the association was attenuated in the high genetic risk group and was no longer statistically significant when adjusting for potential confounders (this attenuation was driven by depressive symptoms in particular). Supplementary analyses excluding AD diagnosed within 5 years of the assessment centre date were consistent with the main models.

These findings may help to clarify previously mixed findings regarding the association between life satisfaction and risk of dementia [6, 8, 9, 10, 11]. Specifically, these findings suggest that this relationship is modified by genetic risk. As such, the lack of consideration of these genetic factors may exaggerate or mask associations, depending on the populations included in samples. These results are consistent with findings for interactions between genetic risk and smoking (another modifiable risk factor for dementia) on dementia outcomes. Specifically, three studies have shown that the relationship between smoking status and dementia is modified by genetic risk for dementia, whereby the association exists only in those with lower genetic risk [14, 15, 16]. However, findings are not consistent with research showing an interaction between depression and APOE genotype on risk of Alzheimer's disease, in which strongest associations were observed in participants with the APOE‐ε4 allele [19].

There are several potential mechanisms which may underlie observed associations between life satisfaction and risk of developing AD. Specifically, this relationship may operate through a range of key lifestyle or biological pathways. For example, people with higher levels of life satisfaction are more likely to engage in healthy lifestyles, for example, regular physical activity and non‐smoking [25], which in turn are associated with reduced risk of dementia [4]. In addition, life satisfaction is related to social engagement and lower levels of loneliness [25], which are also modifiable risk factors for dementia [4]. Beyond this, chronic psychological stress and lower levels of wellbeing are associated with hypothalamic‐pituitary‐adrenal (HPA) axis dysregulation, as well as higher cortisol levels [26], which are associated with neurodegenerative processes [27].

However, these findings show that this relationship does not operate equally across the population, since higher genetic risk may weaken the protective effect of life satisfaction on the risk of AD. There are several plausible explanations for this. First, life satisfaction is associated with healthier lifestyles [25], but in people with higher genetic risk, these lifestyle factors may not be sufficient to counteract the impact of genetics on the risk of AD. Second, there may be important temporal considerations which can explain these findings. For example, it is possible that life satisfaction may have stronger protective effects before or earlier in the disease process (e.g., prior to neuropathological changes associated with AD). People with higher genetic risk may have earlier onset and faster progression of AD than those with lower genetic risk, shortening the window of time in which life satisfaction can exert any potential influence. However, to our knowledge, these hypotheses have not yet been formally tested.

Findings should be interpreted with reference to previous research on AD risk within the UK Biobank. In particular, studies have shown that psychological factors such as depression and personality traits (particularly neuroticism) are associated with an increased risk of AD [28, 29]. Life satisfaction is closely related to these traits, with prior UK Biobank research showing inverse associations between life satisfaction and both neuroticism [30] and depression [31]. This may suggest shared or overlapping pathways between personality traits, depressive symptoms, life satisfaction, and neurodegenerative conditions such as AD. Although depressive symptoms were adjusted for in these analyses, personality traits such as neuroticism were not included in models.

### 4.1 Limitations and Future Research

The study is subject to several limitations which should be considered when interpreting findings. While the study adjusts for several demographic factors, the results may not be generalisable to populations outside the studied sample, particularly if there are significant differences in genetic, environmental, or cultural factors influencing AD risk. In addition to this, life satisfaction was measured at a single time point, which may not fully capture its dynamic nature over time. Life satisfaction can fluctuate, particularly in response to life events and health changes. As such, a single measure at one time point may not reflect long‐term wellbeing. Although previous research has shown that single measures of life satisfaction can have predictive validity for long‐term health outcomes [32], it is important to acknowledge that they may not fully capture long‐term satisfaction and the potential cumulative impact on the risk of AD. Longitudinal tracking of life satisfaction could provide a more accurate picture of its relationship with AD and other neurodegenerative conditions. Further, despite the associations found, the study cannot establish causality. The relationship between life satisfaction, genetic risk, and AD is likely complex, and other mediating or moderating factors may be involved that were not fully explored in this analysis.

The study is also limited by a lack of ethnic diversity in the sample, with only 3.58% of the sample belonging to minoritised ethnic groups. This limits the generalisability to the wider UK population, particularly given known disparities in dementia risk and access to dementia diagnosis within minoritised ethnic communities.

It is also possible that prodromal or preclinical AD present but undiagnosed at the time of baseline may have impacted results. Although this was accounted for by excluding people who were diagnosed with AD within 1 year (in main analyses) and 5 years (supplementary analyses) of the baseline, this does not completely eliminate the possibility of reverse directionality, especially given that the prodromal period of AD can last for many years [33]. Future research could aim to test these interactions over a longer period, ideally over the life course. In addition, the outcome measure of AD was based on clinical diagnosis data, with only approximately 1.63% of the sample receiving a recorded diagnosis over the follow‐up period. Recent estimates suggest that only 65%, 54% and 29% of dementia cases are diagnosed in England, Wales and Scotland, respectively [34]. As such, some AD cases may have been missed in this study. Future research should replicate these findings using alternative measures of AD that do not rely on clinical diagnosis. Further, measures of other domains of wellbeing that have been consistently associated with risk of dementia in previous research, such as the eudaemonic construct of purpose in life [6, 35], were not available in these data. Future research should aim to test interactions between other domains of wellbeing and genetic predisposition on AD. Finally, future research could conduct this research using alternative indicators of genetic risk for AD (e.g., APOE genotype).

### 4.2 Implications

These findings have important implications for improving risk prediction for AD by considering the potentially complex interplay between modifiable risk factors (such as life satisfaction) and genetics on risk. These results suggest that future multi‐domain interventions designed to reduce risk or delay the onset of AD should consist of targeted and personalised prevention strategies, considering key factors such as genetic risk for AD. However, regardless of genetic predisposition, improving life satisfaction (and other domains of wellbeing) is an important target for research, clinical practice and policy to improve overall life quality.

## 5 Conclusion

In conclusion, these results highlight the interplay between psychological factors (specifically life satisfaction) and genetic predisposition on AD incidence. Specifically, associations were observed between life satisfaction and risk of AD onset in people with lower but not higher genetic risk scores. These findings suggest that the influence of life satisfaction on AD risk is not uniform across all individuals. Instead, this relationship is dependent on a person's genetic susceptibility to AD, meaning that life satisfaction may have a stronger or weaker effect on AD risk depending on their genetic risk profile. The findings clarify mixed previous research on this topic and may contribute to more tailored approaches to AD prevention in the future.

## Ethics Statement

UK Biobank has approval from the North West Multi‐centre Research Ethics Committee (MREC) as a Research Tissue Bank (RTB) approval.

## Consent

All UK Biobank participants provided written informed consent.

## Conflicts of Interest

The authors declare no conflicts of interest.

## Supporting information

Supporting Information S1

## Data Availability

Data are available upon application to the UK Biobank team.
