# Supplementary material for: Understanding Interactions Between Life Satisfaction and Genetic Predisposition on Risk of Alzheimer's Disease up to 14 Years Later: Findings From the UK Biobank
Source: Int J Geriatr Psychiatry. 2025 Jun 29;40(7):e70120. doi: 10.1002/gps.70120 (PMC12206470; doi:10.1002/gps.70120)
Supplement: Supplementary file 1 — Supporting Information S1 [file GPS-40-e70120-s001.docx]

**Supplementary Materials**

**Supplementary Figure 1:** Sample selection.

**Supplementary Table 1:** Missing data analysis.

**Supplementary Table 2:** Raw comparisons between people who did and did not develop AD.

**Supplementary Table 3:** Associations between life satisfaction and AD PRS with incidence of AD diagnosis, excluding follow up <5 years.

**Supplementary Table 4:** Interactions between life satisfaction with AD PRS on incidence of AD diagnosis, excluding follow up <5 years.

**Supplementary Figure 1:** Sample selection.

**
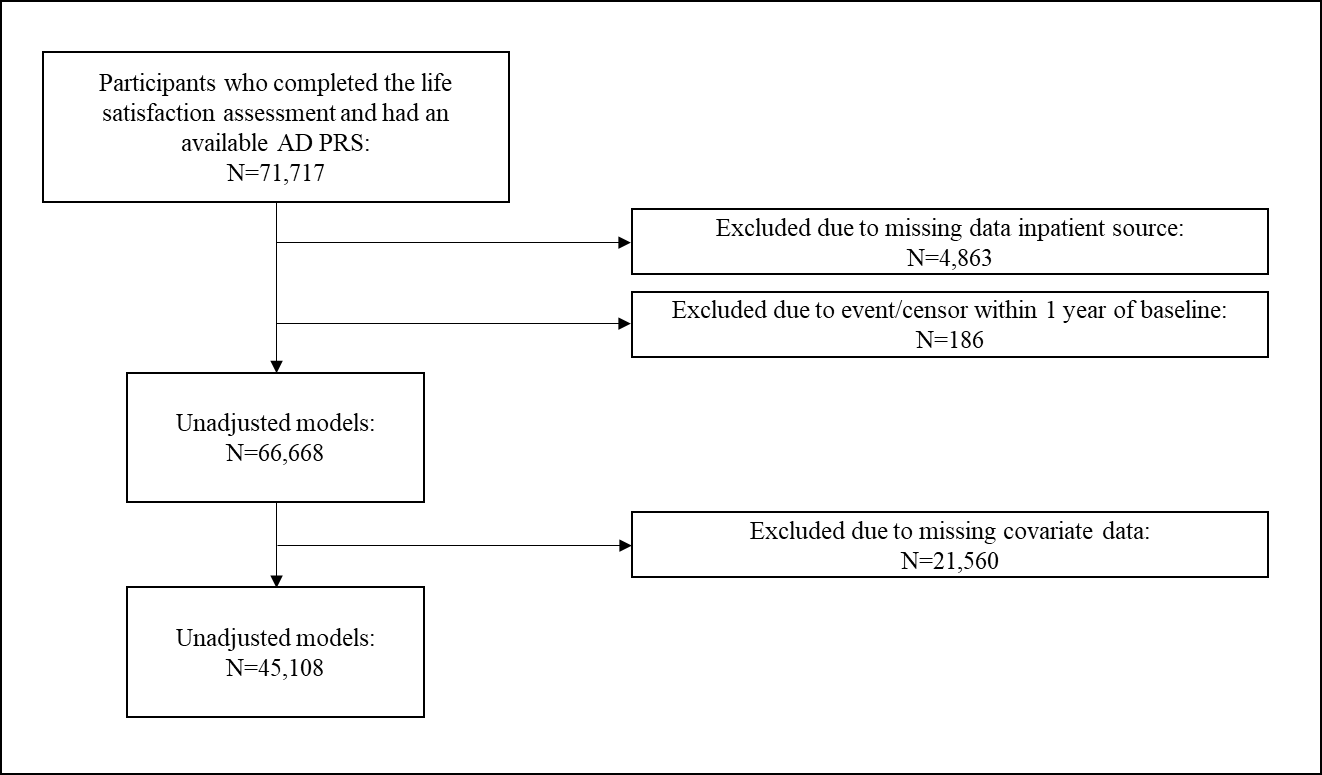
**

**Supplementary Table 1:** Missing data analysis.

| **Characteristic** | **Sample with missing data (N=49,175)** | **Sample with complete data (N=45,108)** | **Comparison  (t-test or chi-square)** |
| --- | --- | --- | --- |
| Age, Mean (SD) | 63.97 (2.80) | 64.31 (2.79) | -18.71 (94,281), <.001 |
| Sex, N (%) |  |  |  |
| Female | 25,460 (51.77) | 24,218 (53.69) | 34.60 (1), <.001 |
| Male | 23,715 (48.23) | 20,890 (46.31) |  |
| Ethnicity, N (%) |  |  |  |
| White | 45,911 (94.60) | 43,491 (96.42) | 203.60 (5), <.001 |
| Mixed | 198 (0.41) | 150 (0.33) |  |
| Asian/Asian British | 1,229 (2.53) | 672 (1.49) |  |
| Black/Black British | 634 (1.31) | 486 (1.08) |  |
| Chinese | 121 (0.25) | 77 (0.17) |  |
| Other | 439 (0.90) | 232 (0.51) |  |
| Deprivation, Mean (SD) | -1.38 (2.96) | -1.42 (2.82) | 2.25 (94,184), .02 |
| Age Left Education, Mean (SD) | 16.46 (2.30) | 16.50 (2.32) | -2.13 (66,563), .03 |
| Depression, N (%) |  |  |  |
| Not at all | 37,126 (82.62) | 36,765 (81.50) | 27.82 (3), <.001 |
| Several days | 6,375 (14.19) | 6,679 (14.81) |  |
| More than half the days | 906 (2.02) | 1,095 (2.43) |  |
| Nearly every day | 529 (1.18) | 569 (1.26) |  |

**Supplementary Table 2:** Raw comparisons between people who did and did not develop AD.

|  | **Alzheimer's disease** | | **Difference** |
| --- | --- | --- | --- |
|  | **No (N=44,373)** | **Yes (N=735)** | **t (df), *p*** |
| Life satisfaction | 3.60 (0.55) | 3.53 (0.56) | 3.26 (45106), .001 |
| AD PRS | 0.02 (0.97) | 0.99 (1.21) | -26.73 (45106), <.001 |

**Supplementary Table 3:** Associations between life satisfaction and AD PRS with incidence of AD diagnosis, excluding follow up <5 years.

|  | **Model 1: Unadjusted N=64,985** | **Model 2: Fully adjusted* N=43,917** |
| --- | --- | --- |
| Life Satisfaction | 0.82 (0.73-0.92) <.001 | 0.84 (0.73-0.98), 0.02 |
| AD PRS | 2.22 (2.10-2.34), <.001 | 2.26 (2.12-2.41), <.001 |
| Age | - | 1.25 (1.21-1.29), <.001 |
| Sex |  |  |
| Female | - | *REF* |
| Male | - | 1.11 (0.96-1.30), 0.17 |
| Ethnicity |  |  |
| White | - | *REF* |
| Mixed | - | 1.52 (0.57-4.07), 0.41 |
| Asian/Asian British | - | 0.94 (0.50-1.78), 0.85 |
| Black/Black British | - | 1.49 (0.79-2.83), 0.22 |
| Chinese | - | 0.81 (0.11-5.81), 0.84 |
| Other | - | 0.49 (0.12-1.98), 0.32 |
| Deprivation | - | 1.04 (1.01-1.07), .004 |
| Age Left Education | - | 0.98 (0.95-1.02), 0.30 |
| Depression |  |  |
| Not at all | - | *REF* |
| Several days | - | 1.17 (0.95-1.45), 0.14 |
| More than half the days | - | 1.46 (0.94-2.26), 0.09 |
| Nearly every day | - | 0.94 (0.44-2.01), 0.88 |

*Proportional hazards assumption not violated: χ2=11.50, df=14, *p*=.65

**Supplementary Table 4:** Interactions between life satisfaction with AD PRS on incidence of AD diagnosis, excluding follow up <5 years.

|  | **Model 1: Unadjusted N=64,985** | **Model 2: Fully adjusted N=43,917** |
| --- | --- | --- |
| Life Satisfaction | 0.74 (0.64-0.86), <.001 | 0.71 (0.59-0.85), <.001 |
| AD PRS | 1.56 (1.11-2.18), 0.01 | 1.19 (0.79-1.78), 0.40 |
| Life Satisfaction X AD PRS | 1.10 (1.01-1.21), 0.04 | 1.20 (1.07-1.34), .002 |
| Age | - | 1.25 (1.21-1.29), <.001 |
| Sex |  |  |
| Female | - | *REF* |
| Male | - | 1.11 (0.96-1.30), 0.17 |
| Ethnicity |  |  |
| White | - | *REF* |
| Mixed | - | 1.49 (0.55-3.99), 0.43 |
| Asian/Asian British | - | 0.95 (0.51-1.80), 0.89 |
| Black/Black British | - | 1.48 (0.78-2.81), 0.23 |
| Chinese | - | 0.78 (0.11-5.55), 0.80 |
| Other | - | 0.49 (0.12-1.97), 0.31 |
| Deprivation | - | 1.04 (1.01-1.07), .004 |
| Age Left Education | - | 0.99 (0.95-1.02), 0.30 |
| Depression |  |  |
| Not at all | - | *REF* |
| Several days | - | 1.16 (0.94-1.44), 0.16 |
| More than half the days | - | 1.45 (0.93-2.24), 0.10 |
| Nearly every day | - | 0.86 (0.40-1.84), 0.70 |
